# Outlines of Anatomy

**Published:** 1902-04

**Authors:** 


OUTLINES OF ANATOMY. A Guide to the Methodical Study of the Human Body in the Dissecting Room. By EDMUND W. HOLMES, A. B., M. D., Demonstrator of Anatomy, University of Pennsylvania (1892–1901); Surgeon to the Methodist-Episcopal Hospital, etc. Second edition. Lancaster, Pa.: The New Era Printing Co. 1902.

This little work already in its second edition is intended for the use of students in the dissecting room. As an elementary treatise and working guide at the dissecting table, it will prove invaluable. Perhaps the most difficult task for an author is to epitomise a subject for the student, and only those who have had the experience of Dr. Holmes are in a position to place before him the helpful hints and concise directions which this small work contains.

In the preface of the second edition, the author says that the work was prepared "in an endeavor to introduce method and discipline into the dissecting room and to thus revivify, if possible, the interest in practical anatomy." Those who have had the previlege of working under Dr. Holmes and know the enthusiasm he instilled into his work will especially appreciate this small work.

The order of sequence in the dissection is that usually followed, beginning with the dissection of the upper extremity, but an extremely useful novelty is found in the division of the work for the benefit of the student into days. Thus the work for the first day on the upper extremity consists of the dissection of the superficial fascia, the superficial nerves, the cephalic vein, etc., and the structures which are exposed upon the first division of the integument. On the second day the dissection of the pectoral muscles follows, and on the third day the axillary space is entered. In this way the amount of work the student should accomplish on each day is definitely indicated and he is prevented from undertaking too much, while at the same time he is informed what should constitute a fair amount of work for a given time.

A number of useful diagrams especially a series showing the folds of the peritoneum, printed in two colors, greatly adds to the value of the work. Taken in conjunction with the larger works on anatomy, these "outlines" should find their way to every dissecting room in the country. H.R.G.

